# Unilateral neglect post stroke: Eye movement frequencies indicate directional hypokinesia while fixation distributions suggest compensational mechanism

**DOI:** 10.1002/brb3.1170

**Published:** 2018-12-12

**Authors:** Kjersti M. Walle, Jan E. Nordvik, Frank Becker, Thomas Espeseth, Markus H. Sneve, Bruno Laeng

**Affiliations:** ^1^ Department of Research Sunnaas Rehabilitation Hospital Oslo Norway; ^2^ Department of Psychology University of Oslo Oslo Norway; ^3^ Institute of Clinical Medicine University of Oslo Oslo Norway

**Keywords:** divided attention, eye‐tracking, Multiple Object Tracking, neglect, nonspatial attention, spatial attention

## Abstract

**Introduction:**

Eye movements and spatial attention are closely related, and eye‐tracking can provide valuable information in research on visual attention. We investigated the pathology of overt attention in right hemisphere (RH) stroke patients differing in their severity of neglect symptoms by using eye‐tracking during a dynamic attention task.

**Methods:**

Eye movements were recorded in 26 RH stroke patients (13 with and 13 without unilateral spatial neglect, and a matched group of 26 healthy controls during a Multiple Object Tracking task. We assessed the frequency and spatial distributions of fixations, as well as frequencies of eye movements to the left and to the right side of visual space so as to investigate individuals’ efficiency of visual processing, distribution of attentional processing resources, and oculomotoric orienting mechanisms.

**Results:**

Both patient groups showed increased fixation frequencies compared to controls. A spatial bias was found in neglect patients’ fixation distribution, depending on neglect severity (indexed by scores on the Behavioral Inattention Test). Patients with more severe neglect had more fixations within the right field, while patients with less severe neglect had more fixations within their left field. Eye movement frequencies were dependent on direction in the neglect patient group, as they made more eye movements toward the right than toward the left.

**Conclusion:**

The patient groups’ higher fixation rates suggest that patients are generally less efficient in visual processing. The spatial bias in fixation distribution, dependent on neglect severity, suggested that patients with less severe neglect were able to use compensational mechanisms in their contralesional space. The observed relation between eye movement rates and directions observed in neglect patients provides a measure of the degree of difficulty these patients may encounter during dynamic situations in daily life and supports the idea that directional oculomotor hypokinesia may be a relevant component in this syndrome.

## 1 INTRODUCTION

Unilateral spatial neglect is a neurological syndrome characterized by attention difficulties that affect the person's ability to perceive or respond to information in the space contralateral to the brain injury (Heilman & Valenstein, 1979; Mesulam, 1981, 1999; Verdon, Schwartz, Lovblad, Hauert, & Vuilleumier, 2010). While damage to either hemisphere may lead to symptoms of neglect in the acute phase after a stroke (Stone, Halligan, & Greenwood, 1993), right hemisphere (RH) injuries more commonly lead to severe and lasting neglect, which has led to more studies investigating neglect as result from RH injuries (Corbetta & Shulman, 2011; Heilman & Valenstein, 1979; Mesulam, 1981; Parton, Malhotra, & Husain, 2004; Stone et al., 1993). Unilateral spatial neglect often derives from brain injuries in the inferior parietal lobule at the right temporo‐parietal junction (Himmelbach, Erb, & Karnath, 2006; Vallar, 1998), but the syndrome has also been linked with other unilateral lesions to subcortical and cortical regions (Karnath, 2001), like the frontal eye fields in monkeys (Kennard, 1939) as well as in humans (Guitton, Buchtel, & Douglas, 1985) and with damage to the dorsolateral premotor and medial frontal regions (Husain & Kennard, 1996). The syndrome is typically not attributable to damage to the sensory or motoric system primarily (Behrmann, Ghiselli‐Crippa, & Dimatteo, 2001; Heilman, Valenstein, & Watson, 2000); instead, several higher level cognitive accounts have been proposed to explain the mechanisms behind this syndrome. One generally accepted view is that damage to the RH compromises key mechanisms of attention in such a way that the patient fails to distribute or orient attention, particularly in the field contralateral to the brain injury (Behrmann, Watt, Black, & Barton, 1997; Heilman et al., 2000; Posner & Petersen, 1990; Posner, Walker, Friedrich, & Rafal, 1984, 1987).

Attention can be expressed *overtly*, either by eye movements shifted toward the object of attention so that the fovea typically receives visual input from the attended object, or *covertly* by shifting the “mind's eye,” without actually directing the gaze toward the object of attention (typically keeping fixation on an empty, uninformative position; Findlay & Gilchrist, 2003). However, in typical visual behavior, eye movements and spatial attention are closely associated and it has been shown that attention toward a location typically precedes a saccade made to the same location (Hoffman & Subramaniam, 1995). Attention shifts may play an important role in the programming of subsequent eye movements (e.g., Deubel & Schneider, 1996). In fact, a study by Kowler, Anderson, Dosher, and Blaser (1995) found that perceptual attention toward a target facilitated the launching of saccades. Also, the ability to identify targets was better at the saccadic goal location than elsewhere, and participants were unable to direct a saccade toward one target while accurately making perceptual judgments about another target placed in a different location. Accordingly, eye‐tracking measures (e.g., distribution and properties of fixations and eye movements) would seem fundamental in the research on visual attention, both with normal participants and in its pathological manifestations in neurological patients.

Though neglect has been associated with deficits in attention to stimuli or the general representations of spatial information, a specific form of neglect has been proposed, where the patient experiences a reduced or slowed directional motor control, referred to as *directional hypokinesia*. This syndrome manifests itself as a deficit in planning and initiating, for example, hand movements toward the contralesional hemispace but not for the ipsilesional direction (Behrmann et al., 2001; Heilman et al., 2000). This slowing of directional movement initiation is not necessarily limited to movements performed with the contralesional limb and within the contralesional hemispace, as it may manifest in both the contralesional limb and the ipsilesional limb depending on the direction of the movement per se (Mattingley, Bradshaw, & Phillips, 1992). Therefore, if a patient tries to reach into the contralesional space, similar problems will be met regardless of what hand is used for the purpose (unless the left hand is paretic). *Hypokinesia* can be distinguished from directional *bradykinesia*, which is an impairment of the execution of the motoric action, reducing the velocities or shortening the amplitudes of motoric actions of specific directions (Behrmann et al., 2001; Mattingley, Phillips, & Bradshaw, 1994). *Directional hypokinesia* in neglect is not limited to the planning and initiation of limb movements, as studies of eye movements have also reported direction‐specific impairments in saccadic orienting in neglect patients (Behrmann et al., 2001; Girotti, Casazza, Musicco, & Avanzini, 1983). Thus, although unilateral spatial neglect cannot be primarily explained by fundamental motoric deficits, like gaze paralysis or optic ataxia, directional hypokinesia may constitute an oculomotor component of neglect in the visuospatial and attentional domain.

### 1.1 Stroke, neglect, and eye movements

Interestingly, only a few previous studies have specifically investigated orienting of eye movements in neglect patients. In a seminal study, Girotti et al. (1983) found that neglect patients failed to make saccades toward a target presented in their contralesional hemifield in 25% of trials. Moreover, in the cases where they succeeded in making saccades into the left hemifield, latencies were prolonged and additional multiple small saccades were needed in order to reach the target. Increased saccadic response time was taken as an indication of unilateral hypoarousal, while the complete abolition of a saccadic response was thought to reflect strong inhibition of the arousal response. A more recent neglect study by Behrmann et al. (2001) assessed variables related to the initiation and execution of saccades in left and right directions, while also accounting for the location of targets in left or right space. They found that neglect patients had longer latencies when initiating leftward saccades in the left field compared to rightward saccades in the same field. In the right field however, there were no differences between leftwards and rightwards saccade latencies. Thus, hypokinetic symptoms can be direction‐specific as well as spatially dependent. The same study also investigated durations and velocities of the saccades reflecting the motor execution mechanisms of the saccade more than its attentional component; however, no bradykinetic symptoms were revealed in the neglect patients (Behrmann et al., 2001).

Other studies have investigated the properties of direction‐specific eye movements in neglect patients (Karnath, Niemeier, & Dichgans, 1998; Niemeier & Karnath, 2003). Specifically, Karnath et al. (1998) showed that their group of neglect patients with RH lesions managed, after prompting, to direct their gaze, head, and *eye‐in‐head*, as far toward the left as the control group could (when orienting to peripheral targets by moving the head, an additional eye movement is required, which is referred to as eye‐in‐head). However, when spontaneously exploring the visual field, searching for a target letter, the same patients clearly showed reduced orienting in the left field (Karnath et al., 1998). Niemeier and Karnath (2003) further assessed saccadic eye movements in neglect patients during a free search task and a stimulus‐driven replay condition. In the free search condition, they found no differences in saccadic amplitudes or frequencies concerning direction. However, an exploratory deficit was evident in their neglect patients, as they mostly explored the right field and ignored the left field. In the stimulus‐driven replay condition, the participants were asked to follow a red square taking the same route of eye movements that they themselves had created in the free search condition. In this condition, leftwards directed saccades were reduced in amplitude and they increased in frequency compared to the rightwards directed saccades. Therefore, the location of an object in relation to the position of the eye (eye‐centered position) affected stimulus‐driven orienting mechanisms since saccadic properties were direction‐specific. Because the exploratory deficits were field‐specific instead of direction‐specific, the authors suggested that exploratory saccades were generated in a neural circuit system using different spatial coordinates than stimulus‐driven saccades (Niemeier & Karnath, 2003).

It should be noted that RH stroke patients who lack neglect symptoms in classic diagnostic tests can nevertheless express altered search mechanisms within the left visual field. In fact, Mapstone et al. (2003) reported that both patients with left or right unilateral cerebral lesions, but without any clinical evidence of neglect, were more accurate in detection of targets in their ipsilesional hemispace than in their contralesional hemispace. The patients with RH damage (without neglect) additionally made longer saccades within the left compared to the right hemispace, which is in contrast to what studies of neglect patients have reported, with neglect patients rather showing multiple small saccades within the contralesional space (Girotti et al., 1983). Thus, differences in eye movement properties of RH patients with and without neglect could reflect altered mechanisms of orienting attention or strategies used to overcome attention dysfunctions. Hence, the present assessment of eye movement properties in RH stroke patients (with and without neglect) may therefore help to throw light on these oculomotor mechanisms underlying neglect.

### 1.2 Fixation patterns found in stroke patients with and without neglect

In their seminal study, Behrmann et al. (1997) combined eye‐tracking with a visual search task and tested nine neglect patients, four hemianopic patients, and nine healthy control participants. The neglect patients were found to show a steep gradual increase in the proportion of fixations from the far end of the contralesional left field to the far end of the ipsilesional right field, as well as an atypical tendency to initiate their search ipsilesionally (i.e., within their right field). Remarkably, the hemianopic group showed an opposite pattern of fixations, where fixations were spatially distributed in the inverse manner, with a steep gradual increase in the proportion of fixations from the far end of the ipsilesional right field to the far end of the contralesional left field. The authors suggest the fixational pattern of hemianopic patients reflects their attempt to compensate for their left visual field loss, by orienting their gaze toward the left. In healthy controls, fixations were distributed evenly across the field.

Another standard neglect test, known as the *line bisection* task, where participants are requested to bisect a line at the midpoint, has been used to demonstrate that patients with neglect hardly fixate at the left side of the line, and that they instead keep fixating on a point to the right side of the real center, which they also mark as the subjective midpoint (e.g., Ishiai, 2006).

Neglect patients’ tendency to repeatedly search through items in right locations of space has been shown in several studies using “cancelation tasks” with invisible marking of targets (i.e., marking the targets with the computer mouse, without leaving a trace that it has been registered; Husain et al., 2001; Mannan et al., 2005; Wojciulik, Husain, Clarke, & Driver, 2001). Husain et al. (2001) recorded eye‐tracking measures during such a cancelation task and reported that the neglect patient repeatedly re‐fixated items located on the right and failed to remember having already searched the same locations. The control group on the other hand had very few re‐fixations and would rarely misjudge the re‐fixated item for being a new item. The authors conclude that their neglect patient thus suffers from both a lateral spatial bias, as well as impaired spatial working memory (cf. Tatler, Gilchrist, & Land, 2005)—failing to retain searched locations across saccades—and that the latter impairment may exacerbate the former.

Finally, fewer fixations in the contralesional hemispace compared to the ipsilesional hemispace during a visual search task have also been reported in RH patients without neglect symptoms, whereas left hemisphere patients in the same study showed no such effect (Mapstone et al., 2003), consistent with a dominant role of the RH in spatial orienting abilities.

### 1.3 The present study

The literature reviewed above has in large part focused on neglect patients’ patterns of eye movements and fixations in cancelation/visual search tasks of static stimuli, for example, letters or geometrical shapes. These simple paper‐and‐pencil tests can straightforwardly express neglect by the reduced search performance for targets in left locations.

However, in daily situations, patients need to navigate and search for objects in a dynamic space, where objects move as well as the observers (a situation common in many daily tasks; e.g., moving through a crowded place, watching children play). These “dynamic” features are entirely absent in the standard neglect tests, and it is entirely possible that a patient may fail to show neglect in the standard tests but still reveal abnormalities in tasks that have in fact better “ecological” validity. Moreover, in daily situations, we may need to divide attention over multiple objects (e.g., in traffic situations) and monitor several items moving in space simultaneously. Hence, in the present study, we decided to use the Multiple Object Tracking (MOT) task and concurrently monitor eye movements and fixations as triggered by multiple targets that changed continuously their spatial positions. Intuitively, the dynamic aspect of the MOT task seems useful in assessing attention abilities via eye movements. In fact, by allowing the eyes to move freely it mimics our active perception of dynamic aspects of the environment, providing increased ecological validity compared to most attentional paradigms, which imply a completely static visual world. Additionally, the MOT task is a flexible task that opens for an assessment of several aspects of attention, in particular the effect of mental workload when dividing attention over several objects, by manipulating the number of target items (Alnaes et al., 2014).

The MOT task typically starts by presenting a number of identical objects on a screen. For a short while, a few of these objects (normally between 1 and 5) are highlighted as targets, and the participant will have to remember which ones are targets, as they again turn identical before starting to move unpredictably around on the screen. The participant is requested to attentively keep track of the targets until they stop moving (e.g., Alvarez & Scholl, 2005; Cavanagh & Alvarez, 2005; Scholl, 2009), that is, while fixating a central point. At the end of the trial, the participant is requested either to report whether one finally highlighted object is one of the targets (partial report), or to indicate the final position of all targets tracked (full report).

It is not necessary to enforce fixation at a central point during tracking in MOT in order to show the key effect of cognitive load on accuracy or pupil responses (see Alnaes et al., 2014). With only one target to track, the task simply requires maintaining the focus on the single target and allows a *continuous pursuit* of such an item with the eyes, however, when the load is increased to multiple targets, splitting the attention between multiple foci seems required in order to manage the task (Cavanagh & Alvarez, 2005). Hence, we expect that eye movements may change accordingly, reflecting different tracking strategies. An increased need for spatial information is also expected with increased load, as there are more spatial target locations to keep updated on, and as such, more frequent changes in gaze position are expected to occur, in order to manage the task properly. This would expectedly lead to a higher fixation count in trials with increased workload, in line with studies of airline pilots finding that more precise landings are achieved when pilots make more eye fixations (Kasarskis, Stehwien, Hickox, Aretz, & Wickens, 2001).

To our knowledge, only two studies have investigated neglect with dynamic visual displays (Battelli et al., 2001; Niemeier & Karnath, 2003). One was the study by Niemeier and Karnath (2003) investigating stimulus‐driven eye movements in neglect patients. However, that study only presented participants with a single target stimulus at the time, which followed the participant's own route of eye movements from a previous condition, where the right field had been explored more than the left. The other study investigated three neglect patients performance on the MOT task, but did not include any eye movement measures (Battelli et al., 2001). Neglect patients in Battelli's study were found to struggle with high‐level motion in the left field but not the right field. It should be noted, however, that with few participants in the neglect group findings may not generalize well. The current study made an effort to increase the sample size to more reliably reflect the neglect population, and re‐examine neglect patients target tracking performance in left versus right fields and with single versus multiple target conditions.

Using dynamic stimuli combined with eye‐tracking measures, the present study takes a rather different approach from previous studies of neglect. One goal was to investigate how fixation frequencies can be related to performance accuracy in patients with RH stroke compared to healthy participants. Specifically, we use the patient's scores on a neuropsychological battery of neglect tests—the Behavioral Inattention Test (BIT; Wilson, Cockburn, & Halligan, 1987)—to divide the patient group into two subgroups of patients (with and without neglect), and then, we assess whether the relations between oculomotor parameters (e.g., fixation rates, direction of eye movements) frequency and performance accuracy differ in these groups. If hypokinesia plays a relevant role in neglect, then RH patients with the diagnosis should show more hypokinetic symptoms than RH patients without the neglect diagnosis.

Focusing on subtypes of patients sharing the feature of suffering a lesion in the same RH is also relevant, since the RH has been proposed to play a key role in other aspects of attention, like *intrinsic alertness* (Raz & Buhle, 2006) and *sustained attention* (Robertson, Ridgeway, Greenfield, & Parr, 1997). Thus, it is not unlikely that such attentional mechanisms will be affected to some degree in all RH patients, whether they show spatial inattention by the BIT‐battery or not. Moreover, though the BIT‐battery is primarily considered as a measurement of spatial attention, we can also expect that a large part of the patients with low scores have more reduced “nonspatial” attention abilities, as the degree of nonspatial attention dysfunction, for example, reduced vigilance, has been found to predict severity and convalescence from neglect (Husain & Rorden, 2003).

The severity of neglect (as indicated by the total BIT‐score) may also predict the oculomotor behavior of the patients when tracking objects and, in particular, the patients’ distributions of fixations between left and right fields. Previous studies show that neglect patients can direct their gaze into their left side of space, although at a reduced rate (Behrmann et al., 1997, 2001; Girotti et al., 1983; Karnath et al., 1998; Niemeier & Karnath, 2003). However, a gradual reduction in number of fixations across space is also observed and this has been modeled by assuming an attentional gradient (Pouget, Deneve, & Duhamel, 2002) resulting in decreased likelihood of target detections the more to the left the targets are positioned. An attentional gradient account of neglect (Anderson, 1996) implies that a patient's attention impairment gradually worsens toward the left visual space. Thus, performance should be better in the region of the left space that is closer to the center than in its leftmost periphery.

In the present study, the display was presented centrally and the paths of the targets would extend into both the left and right space, encouraging eye movements in both left and right directions, as well as fixations in both left and right space. It should be noted that the distribution of gaze fixations across fields are determined here in relation to the head position of the participant since the eye‐tracking camera is mounted on the head of the participant and therefore moves in parallel with head movements. Given that participants are free to move their eyes during tracking of targets, an assessment of the proportion of fixations in the left versus right field will reflect the spatial distribution of attention in relation to head‐centered coordinates. Additionally, we can look at the directions of eye movements, as these are relative to the eye’s position at the initiation of the eye movement.

Based on studies suggesting a direction‐specific hypokinetic component in neglect (Behrmann et al., 2001; Girotti et al., 1983), we specifically expect a reduction in the frequency of leftwards directed eye movements compared to rightwards directed eye movements in neglect patients. In the neglect patients, we further expect not only that performance in the left field will be disrupted, but also that the characteristic features of less efficient attention mechanisms will be reflected in the distribution of gaze between the left and right fields. Specifically, we expect neglect to reveal itself through reduced fixations in the left field proportionally to the severity of neglect. Moreover, we expect that less severe neglect may differ by the presence of oculomotor compensational mechanisms, resulting an increase of fixations in the left field compared to the right field.

To sum up our specific predictions are (a) patients are expected to show decreased accuracy in the MOT task as compared to control participants and accuracy scores are expected to decrease with increased attention impairment (reflected by the BIT‐score division of patient subgroups). (b) In correct trials, patients will show increased frequency of fixations compared to controls and this increase is expected to be largest in neglect patients, in proportion with the degree of attention impairment. (c) Increased attentional “load” is expected to affect all groups, decreasing accuracy scores and increasing fixations’ frequencies. (d) In neglect patients, the proportion of fixations is expected to be lower in the left than in the right field, and while this bias is expected to be clearly pronounced in cases of more severe neglect, it is also expected to be reduced with higher BIT‐scores, due to compensational mechanisms emerging with less severe neglect. (e) Neglect patients are expected to have more eye movements directed rightwards than leftwards, as orienting toward the left has been proposed to make up a specific challenge in these patients.

## 2 METHOD

### 2.1 Participants

Twenty‐six unilateral RH stroke patients (20 males and six females) were recruited from Sunnaas Rehabilitation Hospital (Oslo, Norway); 26 healthy control participants (16 males and 10 females) were recruited via contacts and by a request at the Hospital's intranet to participate in this study. Group and subgroup demographics are presented in Table 1 with statistical tests showing that the groups did not differ significantly with regard to distribution of age, sex, handedness or ocular dominance. The individual patient demographics are displayed in Table 2.

**Table 1:** Demographic characteristics

	Patients with neglect (*n* * *=* *13)	Patients without neglect (*n* * *=* *13)	Healthy control participants (*n* * *=* *26)
Sex	4F, 9M	2F, 11M	10F, 16M
Chi‐square	*x* ^2^ (2, *n* = 52) = 2.167, *p* * = *0*.*338		
Ocular dominance	7L, 6R	7L, 6R	13L, 13R
Chi‐square	*x* ^2^ (2, *n* = 52) = 0.077, *p* * *=* *0.962		
Mean age (*SD*)	53.0 (8.9)	52.5 (17.2)	51.6 (12.1)
*T* tests			
Patients with versus without neglect	*t*(17.925) = −0.086, *p* * *=* *0.933		
Patients with neglect versus controls	*t*(37) = −0.365, *p* * *=* *0.717		
Patients without neglect versus controls	*t*(37) = −0.194, *p* * *=* *0.847		
Mean EHI‐score (*SD*)	77.8 (55.0)	63.3 (66.6)	80.1 (37.2)
*T* tests			
Patients with versus without neglect	*t*(24) = −0.607, *p* * *=* *0.550		
Patients with neglect versus controls	*t*(37) = 0.154, *p* * *=* *0.879		
Patients without neglect versus controls	*t*(15.843) = 0.847, *p* * *=* *0.410		
Mean time after stroke (*SD*)	106.2 (88.2)	92.0 (44.4)	*N*/A
*T *test	*t*(24) = −0.520, *p* * *=* *0.608		

*Notes.* Differences between groups were tested with chi‐square or independent samples *t* tests.

EHI‐score = Score from the Edinburgh Handedness Inventory (left handed < −40, ambidextrous = −40 to +40, right handed >+ 40), Time After Stroke is given in number of days, *F* = females, *M* = males, L = left eye, R = right eye.

**Table 2:** Patient demographics

Id	Sex	Age	Ocular dominance	EHI	Handedness	TAS	Etiology	Localization
1	F	63	R	100	R	10.43	BI	FPTO
2	M	36	R	100	R	17.29	ICH	PT BG
3	F	54	R	100	R	11.14	SAH	FTP
4	M	61	R	100	R	16.57	BI	FTP
5	F	60	L	100	R	44.29	BI	FTP
6	M	45	L	100	R	7.43	ICH	FO & BG
7	M	55	R	100	R	16.43	ICH	BG
8	M	61	L	100	R	5	ICH	FP
9	M	49	R	69	R	16.29	ICH	F
10	F	44	L	100	R	3.86	BI	FTP
11	M	42	L	100	R	6.14	BI	FTP
12	M	61	L	100	R	13	ICH	BG
13	M	58	L	100	R	12	BI	FP
14	M	48	R	−100	L	13.71	BI	FP
15	M	20	R	90	R	19.57	BI	FTP
16	M	53	R	100	R	6.57	BI	FTP
17	M	68	L	100	R	25	BI	FPO
18	M	44	L	100	R	10.57	BI	FTP
19	M	26	R	90	R	14.14	ICH	F
20	M	69	L	33	A	16.71	BI	FP
21	M	68	L	100	R	12.14	BI	BG
22	M	64	L	90	R	3.43	BI	BG
23	F	44	L	100	R	7.57	BI	FTP
24	M	40	R	80	R	16	ICH	F
25	M	73	L	100	R	9.71	BI	FPO
26	F	66	R	−60	L	5.29	BI	N.D.

*Notes.* *M* = Male; *F* = female; Age in years; ocular dominance was obtained with the Miles test (Miles, 1930): L = left dominant eye; R = right dominant eye; EHI = Edinburgh Handedness Inventory score (left handed < −40, ambidextrous = −40 to +40, right handed >+ 40); TAS = time after stroke in weeks at inclusion; L = left handed; R = right handed; A = ambidextrous; ICH = intracerebral hemorrhage; BI = brain infarct; SAH = subarachnoidal hemorrhage; BG = basal ganglia; *F* = frontal; P = parietal; T = temporal; O = occipital; N.D. = no data.

The six conventional subtests of the BIT (Wilson et al., 1987) were completed by all patients. Thirteen patients with a total score at or below 129 were accordingly diagnosed with unilateral neglect (in accordance with the BIT manuals cutoff score for neglect), while the remaining 13 patients with higher scores were classified as non‐neglect patients (see Table 3). A Snellen chart (Snellen, 1862) was used to test the participant's visual acuity before participation to make sure all participants had the proper visual acuity to perform the task. All participants had normal or corrected to normal vision during testing.

**Table 3:** Behavioral inattention test scores

Id	Cancelation tasks			Drawing tasks		Line Bisection score	Total BIT‐score	Attention impairment
	Line crossing	Letter cancellation	Star cancellation	Copying tasks	Representational drawings			
1	12	9	12	0	1	2	44	USN
2	15	9	16	3	1	0	50	USN
3	18	11	21	3	2	0	55	USN
4	0	12	18	2	0	6	78	USN
5	18	15	24	2	0	8	83	USN
6	1	14	17	3	2	0	83	USN
7	2	7	3	0	N/A	0	86	USN
8	5	11	10	2	1	1	92	USN
9	0	3	9	3	0	6	95	USN
10	5	17	10	3	2	4	103	USN
11	0	3	2	3	3	3	114	USN
12	4	10	1	4	3	9	119	USN
13	0	0	5	3	3	9	128	USN
14	0	−1	−3	2	2	7	135	MAI
15	0	0	−1	3	3	7	136	MAI
16	0	−1	1	4	2	8	138	MAI
17	0	0	2	3	1	7	139	MAI
18	0	−1	1	4	3	8	141	MAI
19	1	−1	0	4	3	9	142	MAI
20	−1	−1	0	3	2	9	142	MAI
21	0	0	0	4	N/A	9	143	MAI
22	0	−3	0	4	3	9	143	MAI
23	0	1	0	4	3	9	143	MAI
24	0	0	1	4	3	8	144	MAI
25	0	0	0	4	3	9	144	MAI
26	0	0	0	4	3	8	145	MAI
USN mean of total BIT‐scores							86.92	
USN *SD*							26.04	
MAI mean of total BIT‐scores							141.15	
MAI *SD*							3.18	

*Notes.* Neglect scores in the conventional subtests of the Behavioral Inattention Test. Cancelation tasks: Line Crossing, Letter Cancellation and Star Cancellation: The score report of the difference in number of targets detected in the left and right hemispace, and this value represents the number of targets detected in the left hemispace subtracted from the number of targets detected in the right hemispace; Figure/Shape Copying: 4 is the maximum score; Rep. = Representational Drawing: 3 is the maximum score; Line Bisection: Three horizontal lines were presented on a sheet of paper, one in the left side, in the center and in the right side of the paper. Patients were instructed to mark the center of each line. For each response that did not deviate more than 12.75 mm from the true center of the line 3 points were given, deviations of <19 mm qualified for 2 points, and deviations <25.5 mm gave 1 point. In total, 9 points was the maximum score for all three lines; Total BIT: This score sums up all the targets detected (across both hemispaces) in the cancelation tasks, as well as the test scores of all the drawing tasks and the line bisection task; Attention Impairment: depending on the total BIT‐score, attention impairment was described as MAI = No neglect but possible mild attention impairments for scores over 129 and USN = Unilateral Spatial Neglect for scores at or under 129.

Patients were included in the study if their stroke occurred <12 months prior to study inclusion, and if they had no history of neurological injury previous to the stroke. Two patients who had prior strokes to the same hemisphere were allowed inclusion in the study. No participant (patient or control) with severe cognitive deficits such as dementia, or a history of severe psychiatric disorders or substance abuse, was included in the study. All participants gave informed consent in writing before participation, and no compensation was offered for partaking. The study was examined and approved by The Regional Ethical Committee for the South East of Norway (2011/1589, REK‐sør‐øst).

### 2.2 Materials

The severity of each patient's visual attention impairment was assessed through a set of neglect tests from the BIT (Wilson et al., 1987). These tests included the tests of Line Crossing, Letter Cancellation, Star Cancellation, Figure and Shape Copying, Line Bisection, and Representational Drawing from the BIT (Wilson et al., 1987).

In addition to this, we assessed each patient's visual perimetry with the Friedman Visual Field Analyser 2 (Clement Clarke International Ltd.) to reveal the presence of visual field deficits in the patient group. The perimetry test provides an assessment of the participant's light sensitivity in different locations of the visual field while the eye(s) are fixated at the center of the screen. If a substantial number of neighboring targets are not detected in the left visual field, this could be due to either vision loss or neglect/extinction. It can be difficult to dissociate the neglect and hemianopia diagnoses from each other by use of only a perimetry test; however, we also had neglect tests to include in this evaluation. Since the perimetry test alternates between unilateral and bilateral target presentations, and extinction is a form of neglect where neglect symptoms reveal themselves only when there is strong competition among stimuli (bilateral target presentations), extinction symptoms may be dissociated from symptoms of hemianopia with a perimetry assessment.

Although some patients did show reduced detection of stimuli in the left visual field, which could be consistent with the presence of some scotoma and/or neglect/extinction, no one was diagnosed with hemianopia since they would all respond to some of the visual stimuli in the left visual field.

To consider each individual's overall intellectual functioning after the stroke, we used the Matrix Reasoning test and the Vocabulary test from the Wechsler Adult Intelligence Scale‐Third Edition (WAIS III). The Vocabulary *S*‐score did not reach the norm mean of 10 in any of the patient groups; however, it was well within a standard deviation from the norm mean. Matrix Reasoning scores were also below the norm mean, but both groups were within the normal range of intelligence. Ocular dominance was tested with the Miles test (Miles, 1930), and handedness was tested by use of the Edinburgh Handedness Inventory (EHI) (Oldfield, 1971).

### 2.3 Overall procedure

Each patient had at least three test sessions within 1 week, going through classic pen‐and‐paper neglect tests in the first session, and performing a computerized attention task (MOT) with simultaneous recording of eye movements in the remaining sessions. In addition to the three test sessions, all patients had been assessed with classic psychological tests as part of their hospital assessment and rehabilitation program. We used parts of this assessment to evaluate each participant's general cognitive functioning after their brain injury. The healthy control group was administered the computerized attention task with eye data recording only, as they were expected not to suffer any cognitive inabilities. All tasks were completed in a quiet room with the experimenter present and illumination kept constant during testing. Participants were seated comfortably and asked to sit as still as possible and keep the same body position during testing. The experimenter registered the participants’ verbal responses (yes, no) in each trial by a key press on the computer keyboard.

### 2.4 Setup

Participants were seated approximately 165 cm from a large screen, where the video clips with the dynamic stimuli were projected by use of a NEC NP43 projector. Experiments were created and run using SMI Experiment Center^®^, and monocular data were recorded with a temporal resolution of 50 Hz, by use of a head‐mounted iView eye‐tracking device (HED) (SensoMotoric Instruments, Berlin, Germany). Ocular dominance determined which eye would be tracked during task performance. A 5‐point manual calibration was carried out at the start of each of recording session. By use of infrared light, the eye‐tracker monitored the pupil and corneal reflection and used these measures to determine horizontal and vertical coordinates of gaze position. Finally, SMI BeGaze software was used to extract and export the pupil and response data from the recordings, and the data were then analyzed with SPSS^®^.

### 2.5 Design and presentation of the Multiple Object Tracking task

MATLAB^®^; RRID:SCR_001622 (MathWorks, Natick, MA, USA) and the Psychophysics Toolbox extensions; RRID:SCR_002881 (Brainard, 1997; Kleiner, Brainard, & Pelli, 2007; Pelli, 1997) were used to create the stimuli for the MOT task. The experiment was set up as four blocks of six trial each (adding up to 24 trials), with two blocks being completed in each test session. All participants were tested with displays of two different sizes. Accordingly, each session included one block with a display of 15° visual angle (the total tracking area subtended 15° × 15° visual angle) and one block with a display size of 30° visual angle. This procedure introduced some variability in the stimuli, which seems beneficial with such a challenging task. Half of the participants were randomly selected to start their first session with one display size, while the other half would start out with the other. In each individual's next test session, the order of display sizes was reversed (ABBA/BAAB).

**Figure 1:**
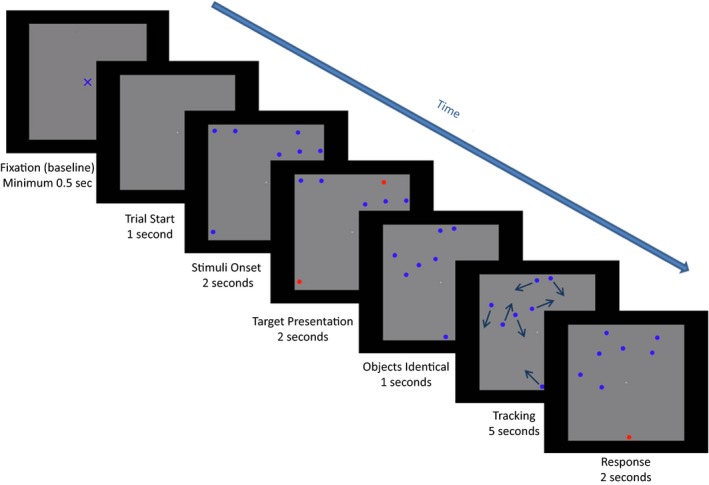
The MOT task. A tracking area empty of objects was first presented, and the participant was asked to fixate at the cross presented at the center of the tracking area. When the participant reported to be ready to start the trial, eight identical circular objects would appear on the screen. Then, one, two, or three objects would change color to red, specifying these to be the targets to track in this trial. Next, targets would turn blue again making them identical to the distractor objects. All objects would then start moving around within the tracking area, and the participant would at their best effort attempt to track the target objects. After 500 ms of tracking the objects would stop moving and one object would turn red. The participant was then asked whether this red object was one of the targets tracked. A “yes” or “no” response was then made and the experimenter registered the response. MOT, Multiple Object Tracking

A centrally presented, gray square constituted the tracking area. A still image of this area empty of objects, but with a blue fixation cross at the center, was presented at the start of every trial. The fixation cross subtended a visual angle of 0.9° or 1.8° in each display version. The participants were instructed to stare at the centered cross and report when they were ready to start the trial. Then, the still image would stay on the screen for an additional 500 ms after which a MOT video clip would start. As the film started, the same tracking area would remain on the screen with the central fixation cross replaced by a small dot. Participants were now free to look anywhere within the tracking area. No objects were presented until 1,000 ms into the film, then eight identical, circular, blue objects with a diameter of 0.55° visual angle in the small display version and a diameter of 1.10° visual angle in the large display version, appeared and remained motionless on the screen. After 1,000 ms, one, two, or three objects changed their color to red, highlighting these as target objects for the present trial. The participant needed to memorize which objects were targets as they all changed back to being blue and thus identical in color to the other circles (distractors) after 2,000 ms. During the next 1,000 ms, the circles would remain still, after which all objects would simultaneously start moving unpredictably, bouncing off each other and off the walls of the tracking area. Objects would move at a velocity of 2.5°/s in trials with a tracking display of 15° visual angle and a velocity of 5.5°/s in trials with a tracking display of 30° visual angle. The participant would track the target objects as they moved among distractors and, after 5,000 ms of tracking, the objects stopped moving. At this point, only one object was highlighted (in red) for the next 2,000 ms and the participant reported whether this highlighted object was one of the targets or not (see Figure 1 for the MOT task event order). In half of the cases, the highlighted object would be a target and equally often it would be a distractor so that the chance of responding correctly was 50%.

Two blocks of six MOT trials were completed in each of the two testing sessions. The three levels of the Load condition were presented twice in each block, once with a target and once with a distractor highlighted at the end of the trial. The order of trials was pseudorandomized and each block would start off with low demands, gradually increasing them and then gradually reducing them toward the end of the block (according to the following sequence of target load: 1‐2‐3‐3‐2‐1). Thus, all levels of Load were presented before any level was repeated, preventing carry‐over effects (e.g., learning effects or fatigue).

At the start of each testing session, the eye‐tracking gear was mounted comfortably on the participant's head. Then, the eye‐tracking system was calibrated, using a 5‐point manual calibration procedure. Each participant had three practice trials. Control questions were asked to ensure that the participant had a full comprehension of the task requirements and particularly the nature of the response (“yes” to a target, “no” if not a target). While the participant could request breaks at any point in between trials, there were longer and systematic breaks between the test blocks.

### 2.6 Data preprocessing

“Fixations” data as well as “eye movements” data were extracted from the eye‐tracking output file using SMI Begaze. SMI BeGaze first detects fixations using a dispersion based algorithm which is considered appropriate from a physiological standpoint. This algorithm searches for fixations as groups of consecutive gaze points within a maximal dispersion of 100 pixels. If such consecutive gaze points are within the maximum dispersion and the time window in which they occur last longer than 80 ms, this is taken as a fixation event. Eye movement events are then computed and derived as the eye movements that occur from one fixation to the next. These eye movements would accordingly include both saccades and smooth pursuit movements. Note that a sampling rate of 50 Hz is not commonly used for investigating eye movements with high precision, as the accuracy of temporal measures tends to be low (Beintema, Loon, & Berg, 2005; Holmqvist et al., 2011, p. 33). However, our goals were merely to detect the occurrence of eye movements and their direction. Nevertheless, due to the low temporal resolution, only a subset of the eye movements was chosen to be included in the analysis. That is, eye movements with amplitudes lower than 1° visual angle were excluded, as these were likely to be microsaccades (Hafed & Clark, 2002; Martinez‐Conde, Macknik, & Hubel, 2000) or glissades (Holmqvist et al., 2011, p. 317). Eye movements over 45° visual angle were also excluded from analysis as 45° has been found to be the neural limit to ocular motility in humans (Worringham, 1991, p. 548). Thus, the calculations of the frequency of eye movements for different conditions included only eye movements between 1° and 45° amplitude.

For analyses on fixations, the number of fixations per trial was extracted from the output of BeGaze software. Separating trials of correct and incorrect responses each participant's average fixation rate per trial was calculated. We then calculated the participant's mean frequency of fixations for each level of the Load condition in correct trials. Finally, as we recorded the participants’ visual fields by mounting a camera on their head, the BeGaze output included spatial coordinates (*X*, *Y*) for each fixation within this recorded visual field. By dividing each patient's visual field by the central coordinates, we were able to separate fixations of the left and right visual field from each other. Participants were instructed to keep their head position throughout the task, and move only their eyes in order to perform the task. The spatial locations of fixations are relative to the head's position, as the camera would move with any head movements. With spatial location of fixations defined, the number of fixations per Visual Field was first counted per trial and then the proportion of fixations being directed to the left Field was calculated per trial (the percentage from the total number of fixations per trial). Finally, each patient's proportion of left field fixations was averaged across trials for correct and incorrect responses separately.

For analyses on eye movements, only trials with correct responses were used. First, eye movements of leftwards and rightwards Directions were teased apart, the eye movement frequencies per Direction were calculated per trial, and then averaged across trials for each participant.

### 2.7 Preliminary analysis

Each participant's accuracy scores per Display size (15° or 30°) were first calculated for a preliminary analysis. One non‐neglect patient had missing data in one of the Display Size conditions and was consequently excluded from the following analysis. A repeated‐measures ANOVA showed no significant effect of Display Size, *F*(1, 48) = 2.223, *p *=* *0.143 (*ns*), and no interaction between Groups and Display Size, *F*(2, 48) = 0.778, *p *=* *0.465 (*ns*). Thus, we collapsed all data across displays for the following analyses.

### 2.8 Analysis

Repeated‐measures ANOVAs were run separately for the three dependent variables of accuracy, fixation frequency and eye movement frequency. If a significant interaction included the between‐subjects factor of Group, separate analyses were run per group to explore the interaction further. Additionally, regression analyses were conducted to assess the ability of BIT‐scores to predict the dependent variable of fixation proportion in the left field (a percentage proportion calculated from the total of fixations of the left and right field). These regression analyses were run separately per patient subgroup. All data analyses were run on IBM SPSS® Statistics; RRID:SCR_002865, version 25.

## 3 RESULTS

### 3.1 Accuracy

A repeated‐measures ANOVA compared accuracy scores across Groups (neglect patients, patients without neglect and healthy controls) and Load conditions (1, 2, or 3 targets being tracked simultaneously).

**Figure 2:**
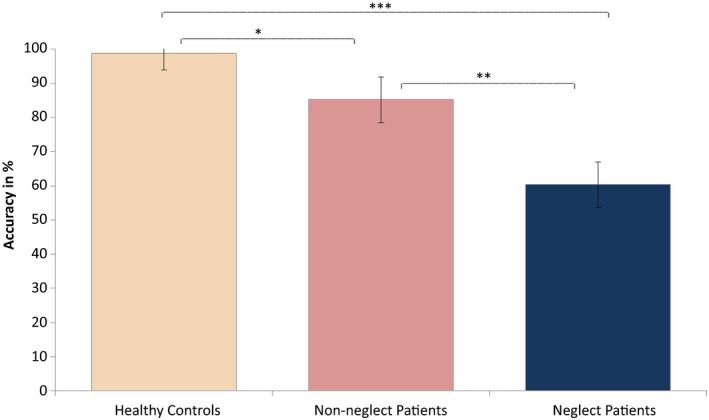
The effect of group on accuracy scores in the MOT task. The figure shows mean accuracy percentage on the MOT task for each group separately. All comparisons of accuracy scores between groups reached significance. Error bars are 95% confidence intervals. Significant between‐group differences are marked with asterisks: *p <* *0.05, **p <* *0.01, and ***p <* *0.001. MOT, Multiple Object Tracking

The main effect of Load only approached significance, *F*(2, 98) = 2.691, *p *=* *0.073, $\eta_{p}^{2}$ = 0.052, with accuracy scores being reduced with higher load. There was a highly significant main effect of Group, *F*(2, 49) = 44.520, *p *<* *0.001, $\eta_{p}^{2}$ = 0.645, as scores decreased with increased attention impairment (see Figure 2). There was no interaction between Load and Group, *F*(4, 98) = 0.786, *p *=* *0.537 (*ns*).

We then examined the significant main effect of Group by running planned paired comparisons between each of the Groups. Since Levene's test was significant, we chose to run the Games–Howell test for this purpose, as it does not assume equal variances. This revealed significantly reduced accuracy scores in (a) neglect patients as compared to controls (mean difference = −0.3834, *SE* = 0.05259, *p *<* *0.001), (b) neglect patients as compared to patients without neglect (mean difference = −0.2488, *SE* = 0.06592, *p *=* *0.003), and (c) patients without neglect compared to healthy control participants (mean difference = −0.1346, *SE* = 0.04065, *p *=* *0.015). Figure 2 illustrates the effect of Group, with controls performing significantly better than both patient groups and patients without neglect performing significantly better than the neglect patients. As shown, neglect patients approached chance performance in their mean accuracy scores across load conditions; however, the chance level of 50% was not included within the group's confidence intervals, suggesting the overall group performance was low but not based on guessing.

### 3.2 Fixation frequency

#### 3.2.1 Interactive effects of Task Performance and Group on fixation frequencies

**Figure 3:**
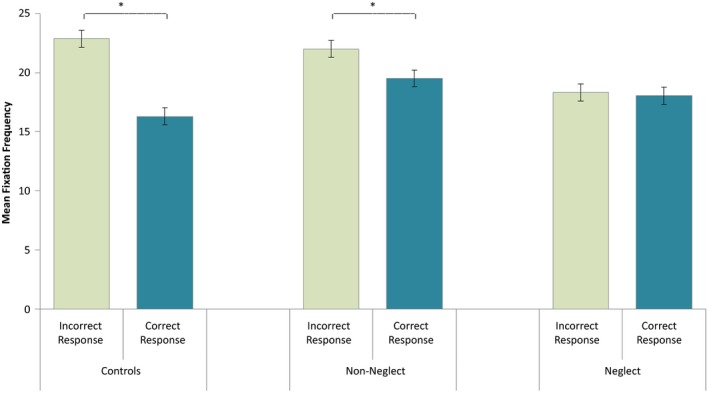
The interaction effect of task performance and group on fixation frequencies in the MOT task. The figure illustrates averages in fixation rates for each group, and for trials of correct and incorrect responses separately. Significant within‐group differences are marked with asterisks. Significant within‐group differences are marked with asterisks: *p <* *0.05, **p <* *0.01, and ***p <* *0.001. 95% confidence intervals were computed according to the formula for within‐subject design of Loftus and Masson (1994). MOT, Multiple Object Tracking

Across all groups, about 86% of trials were correct, while the remaining 14% were incorrect. We performed a repeated‐measures ANOVA to investigate whether fixation frequencies were related to Task Performance (correct and incorrect responses) and whether Groups (neglect patients, patients without neglect and healthy controls) differed on these measures. Fixation frequencies did not deviate significantly from normal on trials of correct responses, *W *(29) = 0.972, *p *=* *0.620, or incorrect responses, *W *(29) = 0.964, *p *=* *0.408. The analysis revealed a significant effect of Task Performance, *F*(1, 26) = 34.496, *p *<* *0.001, $\eta_{p}^{2}$ = 0.570, with increased fixation frequencies for trials with incorrect responses. However, there was also a significant interaction effect between Task Performance and Group, *F*(2, 26) = 10.460, *p *<* *0.001, $\eta_{p}^{2}$ = 0.446, indicating that the effect of Task Performance differed between groups (see Figure 3). There was no significant main effect of Group, *F*(2, 26) = 0.912, *p *=* *0.414 (*ns*).

In order to make a closer assessment of the interaction between Task Performance and Group, separate analyses were run for each Group of participants. These analyses revealed significant effects of Task Performance on fixation frequencies in control participants, *F*(1, 4) = 18.836, *p *=* *0.012, $\eta_{p}^{2}$ = 0.825, and in patients without neglect, *F*(1, 10) = 7.259, *p *=* *0.023, $\eta_{p}^{2}$ = 0.421, as these groups would make more fixations on trials of incorrect responses compared to trials of correct responses (see Figure 3). Neglect patients showed no effect of Task Performance on fixations’ rate, *F*(1, 12) = 0.320, *p *<* *0.582 (*ns*).

#### 3.2.2 Effects of load and group on fixation frequencies

**Figure 4:**
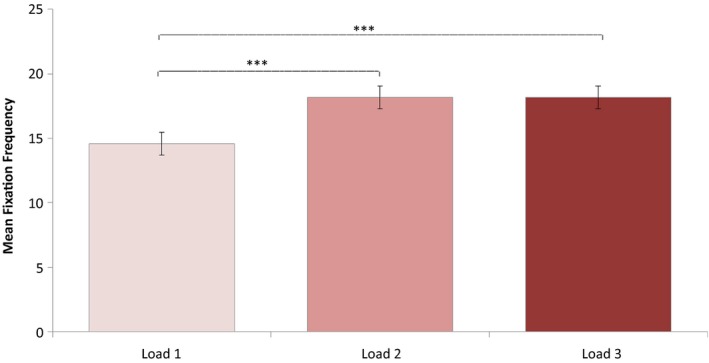
The effect of load (1, 2, or 3 targets) on fixation frequencies in the MOT task. The figure shows the average fixation rate per trial for each condition of load and across all groups. Significant within‐group differences are marked with asterisks: * *p *<* *0.05, **p <* *0.01, and *** *p *<* *0.001. 95% confidence intervals were computed according to the formula for within‐subject design of Loftus and Masson (1994). MOT, Multiple Object Tracking

**Figure 5:**
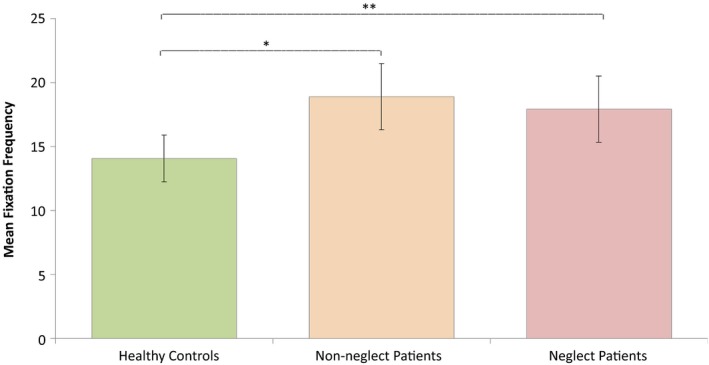
The effect of group on fixation frequencies in the MOT task. The figure shows the mean fixation rate per trial of each group of participants. Error bars are 95% confidence intervals. Significant between‐group differences are marked with asterisks: * *p *<* *0.05, **p <* *0.01, and ***p <* *0.001. MOT, Multiple Object Tracking

Since there was a significant interaction effect of Task Performance and Group on fixation frequencies, the following analyses on fixation frequencies included only trials with correct responses. A repeated‐measures ANOVA was conducted investigating effects of Load (1, 2, or 3 targets being tracked) and Group (neglect patients, patients without neglect and healthy controls) on fixation frequency. Degrees of freedom were corrected by the method of Greenhouse Geisser (*ε* = 0.8). Fixation frequencies did not deviate significantly from normal any of the load conditions: Load 1, *W *(52) = 0.970, *p *=* *0.202; Load 2, *W *(52) = 0.972, *p *=* *0.269; Load 3, *W *(52) = 0.984, *p *=* *0.710. This analysis revealed a significant main effect of Load on fixation frequency, *F*(1.600, 78.416) = 24.355, *p *<* *0.001, $\eta_{p}^{2}$ = 0.332. The contrasts revealed that a Load of two targets lead to significantly more fixations than a Load of only one target, *F*(1, 49) = 33.450, *p *<* *0.001, *r *=* *0.999. Similarly, a Load of three targets lead to more fixations than a load of one target, *F*(1, 49) = 26.480, *p *<* *0.001, *r *=* *0.999 (see Figure 4). In addition, there was a significant effect of Group, *F*(2, 49) = 5.797, *p *<* *0.006, $\eta_{p}^{2}$ = 0.191, showing an increase in fixation rate with more severe attention impairment (see Figure 5). There was no significant interaction between Load and Group, *F*(3.201, 78.416) = 1.811, *p *<* *0.148 (*ns*).

Planned multiple comparisons between Groups revealed significantly higher fixation rates in neglect patients compared to controls (mean difference = 3.8638, *SE* = 1.57974, *p *<* *0.004), and in patients without neglect compared to controls (mean difference = 4.8160, *SE* = 1.57974, *p *<* *0.018). Patients with and without neglect did not differ significantly in their fixation rates (mean difference = −0.9522, *SE* = 1.82412, *p *=* *0.604 (*ns*)). The effect of Group is illustrated in Figure 5.

### 3.3 Proportion of fixations in left and right fields

Simple regression analyses were run separately for each of the two subgroups of patients and for correct and incorrect responses to assess the ability of BIT‐scores to predict proportion of fixations in left versus right field.

#### 3.3.1 Neglect patients

**Figure 6:**
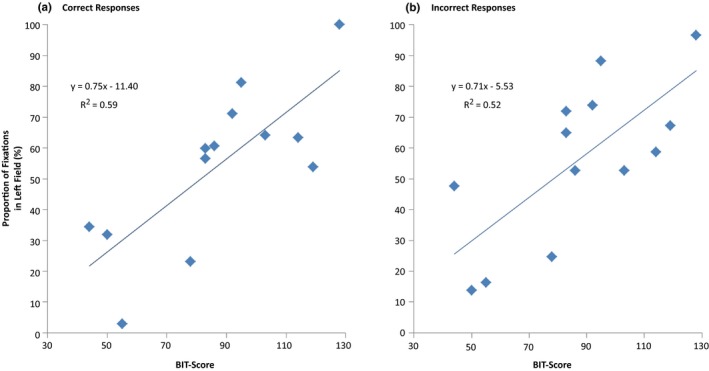
Neglect patients’ fixation distribution between left and right fields. BIT‐scores of neglect patients could be used to predict the fixation distribution between left and right fields in correct (a) and incorrect (b) trials. Diamonds show each patient’s proportion of fixations located in the left field, with the remaining percentage of fixations being located in the right field. The fitted regression line shows the predicted proportion of left field fixations. BIT, Behavioral Inattention Test

In neglect patients, a simple regression with only correct responses revealed that the patients’ BIT‐scores significantly predicted the proportion of fixations in the left field, *F*(1, 11) = 15.694, *p *=* *0.002, with *R *=* *0.767 and *R* ^2^ = 0.588. Thus, BIT‐scores were accordingly estimated to explain 59% of the variance in this group when responding correctly (Figure 6a). A similar simple regression for incorrect responses in this group showed an even stronger relationship between BIT‐scores and fixations’ lateral distribution, *F*(1, 11) = 11.812, *p *=* *0.006, with *R *=* *0.720 and *R* ^2^ = 0.518. BIT‐scores were estimated to explain 52% of the variance in fixation distribution when neglect patients responded incorrectly to the task (Figure 6b). As illustrated in Figure 6, a steep gradient characterizes both the plot for correct (A) and the plot for incorrect (B) responses. Both plots reveal a shift in the spatial bias as the BIT‐scores increase. Neglect patients who scored high on the BIT‐task had a higher proportion of fixations in the left field, while neglect patients who scored low on the BIT‐task had a higher proportion of fixations in the right field.

#### 3.3.2 Patients without neglect

For comparison, simple regression analyses were run with data from patients without neglect as well, similarly assessing whether the BIT‐scores could predict the distribution of fixations across the field. The analyses were also run separately for trials of correct and incorrect responses. Neither of these models reached significance: correct responses, *F*(1, 9) = 0.026, *p *=* *0.875 (*ns*), and incorrect responses, *F*(1, 11) = 0.171, *p *=* *0.687 (*ns*), which is not surprising as these patients were not expected to show an spatial attention bias.

**Figure 7:**
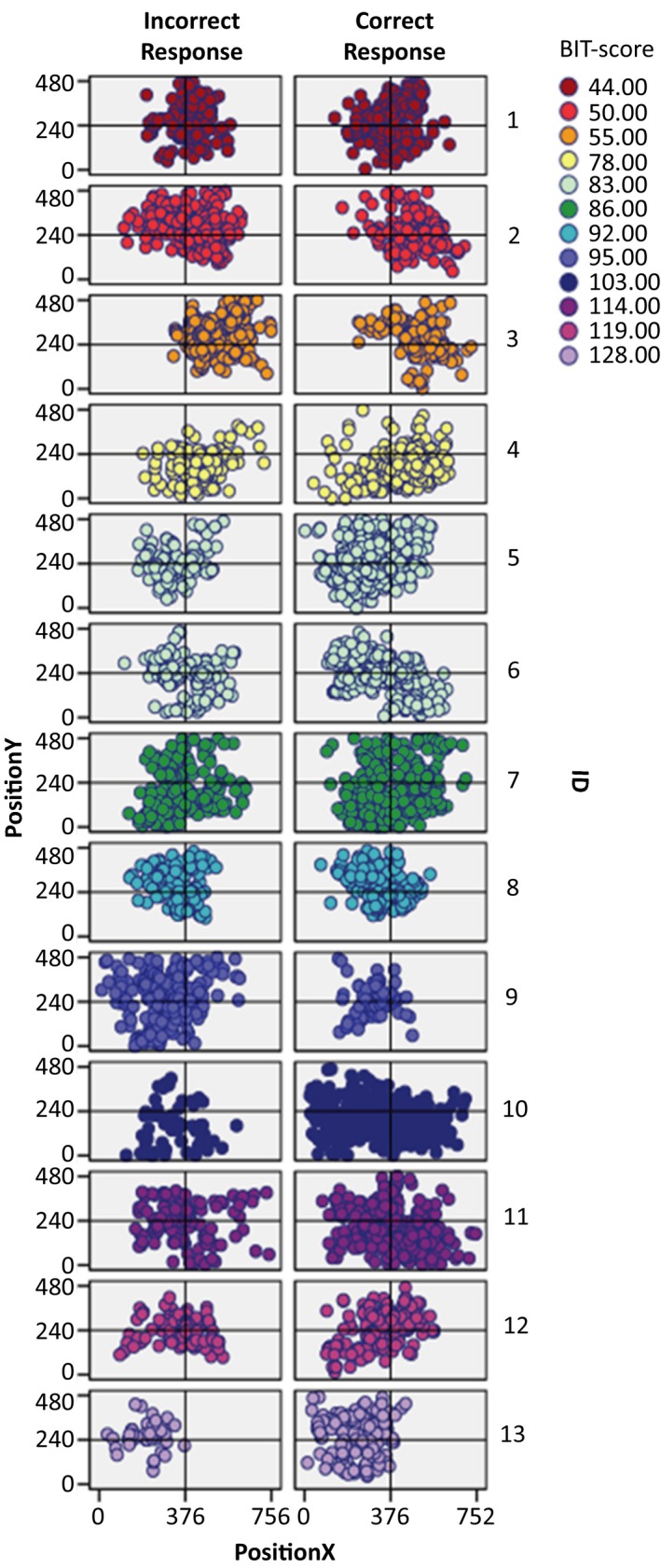
Each neglect patient’s fixation distribution across the field. Each fixation is plotted in accordance with the screen coordinates (752:480 resolution) of the eye‐tracker’s video camera. For each patient, there is one plot showing the fixations of correctly responded trials, and one plot for fixations of incorrectly responded trials. As the BIT‐scores are color coded, the fixations of each patient are colored in accordance with their BIT‐scores. Thus, patients with the same BIT‐score have the same color on their plotted fixations. The patients’ plots are displayed in the order of their BIT‐scores, and as such, one can see that the spatial bias in fixation distribution shifts gradually as the patient BIT‐scores increase in value. BIT, Behavioral Inattention Test

**Figure 8:**
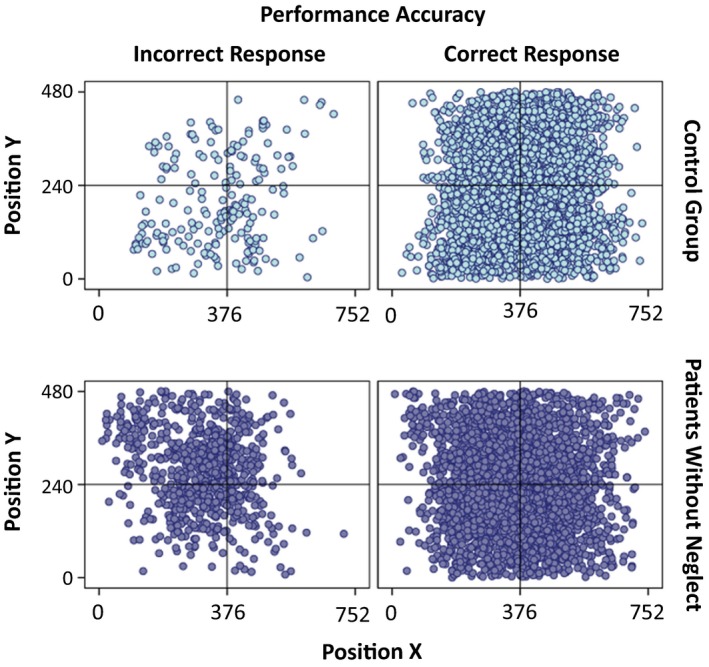
Overall fixation distribution across the field for the healthy control group and patients Without Neglect. Each fixation is plotted in accordance with the screen coordinates (752:480 resolution) of the eye‐tracker’s video camera. For each group, there is one plot showing the fixations made in correctly performed trials, and one plot for fixations made in trials of incorrect responses

In Figure 7, we illustrate the spatial distribution of fixations across the field for each individual neglect patient (the BIT‐score is used as an indication of severity of attention dysfunction, with more severe symptoms, the lower the score). In Figure 8, we show the spatial distribution of fixations across the whole group of patients without neglect as well as the spatial distribution of fixations across the whole control group. In both, fixations from trials of correct and incorrect responses are presented as separate plots.

### 3.4 Eye movement frequency

#### 3.4.1 Interactive effect of eye movement direction and group on eye movement frequencies

**Figure 9:**
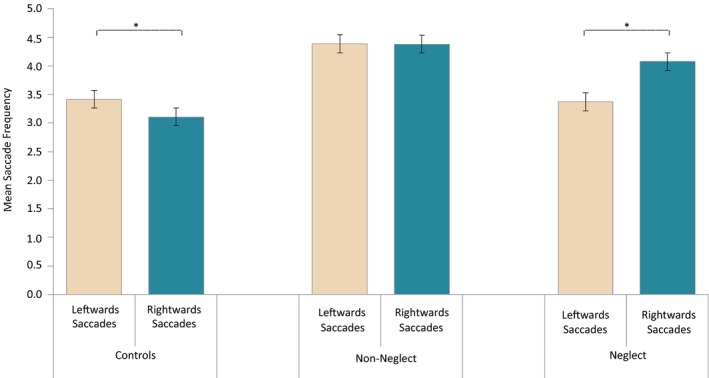
The interaction effect of saccadic direction and group on saccadic frequencies in the MOT task. This figure depicts the average rates of leftwards and rightwards directed saccades and for each group separately. Significant within‐group differences are marked with asterisks: * *p *<* *0.05, **p <* *0.01, and ***p <* *0.001. 95% confidence intervals were computed according to the formula for within‐subject design of Loftus and Masson (1994). MOT, Multiple Object Tracking

The last analyses considered eye movement frequencies in relation to Directions of eye movements (leftwards vs. rightwards) while accounting for effects of Group (neglect patients, patients without neglect and healthy controls). A repeated‐measures ANOVA with eye movement Direction as within‐subjects factor and Group as between‐subjects factor was run, including only trials with correct responses. The analysis revealed a significant interaction between Group and Direction, *F*(2, 49) = 7.095, *p = *0.002, $\eta_{p}^{2}$ = 0.225. As shown in Figure 9, the proportion of rightwards versus leftwards eye movements varied between Groups. No main effects reached significance: Direction, *F*(1, 49) = 1.246, *p = *0.270 (*ns*), and Group, *F*(2, 49) = 2.174, *p = *0.125 (*ns*).

In order to examine the interaction between Direction and Group, analyses were run separately per Group. The analysis of the neglect patients showed a significant effect of Direction, *F*(1, 12) = 6.793, *p = *0.023, $\eta_{p}^{2}$ = 0.361, with more eye movements directed toward the right than toward the left. Remarkably, the analysis of Control participants also revealed a significant effect of Direction, *F*(1, 25) = 7.046, *p = *0.014, $\eta_{p}^{2}$ = 0.220; however, this group showed a pattern opposite to that of neglect patients, making more eye movements directed leftwards than rightwards. The patients without neglect did not show any significant effect of eye movement Direction, *F*(1, 12) = 0.001, *p = *0.980 (*ns*).

## 4 DISCUSSION

As expected from the literature on unilateral neglect, the RH patients diagnosed with the syndrome had the lowest accuracy scores in the present divided‐attention task with dynamic stimuli, or MOT. Also expected, healthy control participants had the best scores, and RH patients without a neglect diagnosis performed better than neglect patients. There was revealed a tendency of lower accuracy with increasing load for all groups. In line with our novel predictions, the frequency of fixations increased across all groups when load was increased above one target and attention had to split between multiple items. All patients revealed increased fixation frequencies compared to controls. However, we did not find conclusive evidence for a stepwise increase in relation with the severity of the attention impairment.

Patients with more severe neglect had, in line with our predictions and previous findings, a lower proportion of fixations placed in the left field. Interestingly, patients diagnosed with neglect but with the less severe symptoms (as indexed by the BIT‐score), had actually more fixations in their left field than in their right field. There was accordingly a gradual shift of the fixation distribution between left and right fields as the BIT‐score increased. As expected, the BIT‐scores did not predict the spatial distribution of fixations in patients without neglect.

Also in accordance with our predictions, eye movement frequencies depended on their direction in neglect patients, as these patients clearly made more eye movements toward the right than toward the left. Interestingly, controls showed the opposite bias, making more eye movements directed leftwards than rightwards, which may reflect a normal greater attentiveness for objects moving within the left visual field (Bosworth, Petrich, & Dobkins, 2012). Patients without neglect did not reveal direction‐specific differences in eye movement frequencies.

### 4.1 Linking efficiency and expertise to group's fixation frequencies

Fixation rates revealed that both groups of RH patients made markedly more fixations compared to the control participants. The fixation rates of the two patient groups, however, did not differ explicitly, and they showed similar increases. Possibly, these are accounted for by the RH brain injury and not by the neglect syndrome per se.

An increase in the frequency of fixations as well as eye movements has previously been associated with decreased efficiency in visual search and information processing in healthy participants (Goldberg & Kotval, 1999). Hence, the present findings indicate that such variables may be also useful for monitoring efficiency levels and changes with neurological patients. An increase in number of fixations may reflect a reduced ability to process information provided with each fixation, and accordingly an increased need for more fixations as less information is processed with each fixation. At least, the present findings suggest that RH patients may need to update visual memory and perceptually sample a greater number of regions of space than is needed in neurologically intact individuals.

Regarding expertise and fixation frequencies, it appears the relation between the two is task‐dependent. That is, several studies have associated a higher level of expertise with fewer fixations and saccades (Krieber et al., 2016; Reingold & Charness, 2005; Reingold, Charness, Pomplun, & Stampe, 2001). These studies in general relate expertise level to fixation counts in chess playing and in reading, which are tasks that rely much on pattern recognition. A different approach was taken by Kasarskis et al. (2001), however, who reported that expert pilots—as compared to novices—make a higher number of fixations on the runway and the cockpit instrument panels, though with significantly shorter durations. Moreover, they found that airplane pilots—regardless of expertise—showed improved performance through more precise landings when their fixation frequencies increased (Kasarskis et al., 2001). It may be that, in highly complex tasks, when there is a need for monitoring large and detailed portions of the visual field, one may benefit from an increase in fixations. Thus, as seen in tasks of pattern recognition (reading and chess playing), a trained eye may recognize the visual patterns more easily with fewer fixations needed, while in complex monitoring tasks, an expert, being highly trained in monitoring large and detailed portions of the field, may be able to efficiently utilize more information from a higher number of fixations.

Hence, we would like to interpret the higher rate of fixations on correct trials in RH patients compared to healthy controls to reflect the RH patients’ generally reduced efficiency in processing of visual input as well as their need for increased perceptual sampling to keep track of the targets. Importantly, even though fixation frequencies in the two patient groups were similar, the accuracy scores did differ between these groups, suggesting the task is more demanding for neglect patients. We point out that an increase in fixation rate could provide the needed visual processing resources to facilitate task performance, at least if these resources are allocated to appropriate locations. While non‐neglect patients may have benefited from distributing these resources in an appropriate manner, the uneven spatial allocation of visual resources shown by neglect patients may explain why this subgroup performed worse, as they may have achieved only small improvements in performance from their increased fixation rates.

### 4.2 Divided attention requires more fixations

An increase in the rate of fixations may also reflect a boost in effort or resources devoted to a task; that is, fixation rates may reflect both the ability to allocate more resources to the task by increasing the amount of input for processing as well as the ability to efficiently extract more information from each fixation. Both mechanisms would be useful when the level of cognitive challenge elicited by a task is increased. In all groups, fixation rates increased when cognitive load increased, suggesting an increase in resource allocation, consistent with the idea that challenging tasks require more frequent and differentiated fixations of gaze (Kasarskis et al., 2001). The rate of fixations may thus be one possible valid indicator of effort or resource investment.

We note that the increase in fixation frequencies occurred when load was increased from a single to multiple targets but when load increased from two to three targets we did not find significant changes in fixation rates. It is possible that tracking a single dynamic target in the MOT task requires keeping a unitary and steady focus on a moving object over time, implying pursuit of the target. However, an increase in load into two targets requires the additional component of *divided attention*, which enables one to split the focus between two items and update information of where they are positioned over time (Alvarez & Cavanagh, 2005; Cavanagh & Alvarez, 2005; Holcombe & Chen, 2013). In contrast, switching from two to three targets would not require switching from a unitary focus to a split mode but only a step change in the amount of split foci. At any rate, divided‐attention mechanism would likely encourage frequent shifts of attention between the multiple targets and accordingly lead to increased rates of fixations.

### 4.3 Spatial distribution of fixations

Earlier studies on fixation distribution across space in neglect patients have shown a clear bias between the fixation frequencies within the right and left space (Behrmann et al., 1997; Ishiai, 2006). This bias in fixation distribution is commonly associated with the neglect of targets in the left hemifield. In the present study, this was certainly true for most patients diagnosed with neglect (and especially so, for those who scored lowest on the BIT‐assessment). For patients with less severe neglect, however, there were indications of what we would like to call a “compensational fixation strategy” prompting an increase in the frequency of fixations within the problematic left hemifield, possibly in order to overcome the lower efficiency in this hemifield. Previous studies have shown that hemianopic patients use similar fixation strategies to help them work around their vision loss (Behrmann et al., 1997). If some neglect patients can be shown to use similar strategies, as seems to be the case for a few of our neglect patients, an analysis of these strategies in future studies may lead to improved recovery or a positive response to relevant interventions. Although there is still a long way to go in gathering empirical evidence for the above points, we believe that an increased understanding of the different components that play a role in attention functioning, as well as how attentional resources are allocated in neglect, may pave the way for targeted interventions toward the relevant mechanisms. Moreover, an increased understanding of these mechanisms may facilitate the refine diagnosis, prognosis, and treatment in other disorders where attention pathologies are also evident.

As expected, in the non‐neglect patient group the BIT‐scores did not predict any spatial bias in fixation distributions. Importantly, BIT‐scores were predictive of fixation distributions in neglect patients, as the spatial bias in fixation distributions was clearly stronger rightwards in patients with more severe neglect. However, the rate of fixations made in left and right fields during tracking could not indicate whether neglect was present or not in an individual, as some patients with less severe neglect actually showed the opposite bias in their spatial distributions of fixation compared to that of the other neglect patients. Instead, the spatial distribution of fixations may provide a useful indication of some neglect patients’ available oculomotor compensatory mechanisms for dynamic attention.

### 4.4 Direction‐dependent eye movement rates

Our results confirm that RH patients with neglect are not necessarily completely akinetic toward the left, as patients in our study succeed in making some leftwards eye movements. At the same time, it was clear that eye movements directed toward the right outnumbered those directed leftwards, even in correctly performed trials. Thus, direction‐specific eye movement properties may provide a valid manner to quantify neglect symptoms, even in those contexts where the patient manages to overcome leftwards inattention.

Posner's theoretical account on mechanisms of attention and neglect (Posner & Petersen, 1990) suggests that orienting mechanisms play an important role in neglect. Most interestingly, the disengagement deficit of neurological patients in the so‐called Posner's cueing task (Posner, Walker, Friedrich, & Rafal, 1987) was determined to be direction‐specific, rather than spatially dependent, since it related to spatial shifts of attention in a contralesional direction regardless of which side of space the target was presented. Hence, it seems relevant to note that also in the present study, a direction‐specific (eye‐centered) deficit was confirmed in our neglect patients, as a reduction in the frequency of leftwards eye movements. Direction‐dependent eye movement rates in neglect patients are also in line with Heilman's theoretical account suggesting that neglect may involve a component of directional hypokinesia (Heilman & Valenstein, 1979). It should be noted, though, that the randomness nature of strokes’ locations and extent may affect a variable number of mechanisms and cognitive functions in different patients and we cannot exclude the possibility that the saccadic patterns could also stem from other affected mechanisms.

It is also interesting to note that the control participants exhibited the opposite pattern to that of neglect patients, with more eye movements directed toward the left than toward the right. Previous studies have also demonstrated a slight but consistent bias of attention toward the left in healthy participants, in other words an opposite spatial bias to that shown in neglect patients (Bowers & Heilman, 1980; Bosworth et al., 2012). Moreover, an initial leftward attention bias found in healthy participants appears to diminish when the participant's alertness is lowered (Dufour, Touzalin, & Candas, 2007). Manly, Dobler, Dodds, and George (2005) showed that when lowering alertness levels sufficiently a leftwards attentional bias can even shift toward a rightward bias. Also in the neglect syndrome, studies have suggested that nonspatial attention mechanisms, like alertness and vigilance, may play significant roles (Posner & Petersen, 1990; Robertson, Mattingley, Rorden, & Driver, 1998; Husain & Rorden, 2003). The neglect syndrome is commonly associated with a decreased general level of alertness (Posner & Petersen, 1990). Moreover, severity of spatial neglect has been found to correlate with severity of nonspatial attention deficits, like impaired vigilance (Husain & Rorden, 2003). Additionally, in neglect patients left spatial inattention have even been shown to improve when alertness levels are increased (Robertson et al., 1998). Since the RH is often associated with alertness mechanisms (Heilman, Schwartz, & Watson, 1978; Robertson & Frasca, 1992; Wilkins, Shallice, & McCarthy, 1987), a possible link between spatial attention biases and nonspatial alertness mechanisms could provide an explanation for the opposite biases found in the neglect and control group. It could also explain the non‐biased pattern found in non‐neglect patients, as these may be expected to show reduced alertness due to the RH stroke, but not sufficiently to cause neglect symptoms. This would fit with the idea that RH stroke may reduce the functioning of alertness networks in the brain (Robertson & Frasca, 1992) and that neglect severity is linked with degree of sustained alertness (vigilance) impairment (Robertson et al., 1997; Hjaltason, Tegner, Tham, Levander, & Ericson, 1996).

It should be noted that although previous studies have suggested reduced mechanisms for shifting attention leftwards in neglect (Behrmann et al., 2001; Girotti et al., 1983), one study by Niemeier and Karnath (2003) actually presented the opposite directional patterns in eye movement rates of neglect patients. An important note though is that the present results are based on the frequencies of eye movements larger than 1 degree in amplitude. Thus, the dissociation between results of the present study and that of Niemeier and Karnath (2003) could possibly be explained by the inclusion of smaller, correctional saccades in the earlier study. If larger saccades toward the right tend to outnumber larger saccades toward the left (as shown in the present study), there could also be a higher frequency of smaller correctional saccades toward the left than of those toward the right (as shown in the earlier study). Opposite biases between larger and smaller eye movements could thus tip results of the two studies in different directions. As we do not investigate the smaller saccades specifically in the current work, this cannot be stated from the present results. However, future studies using eye‐tracking equipment with higher temporal resolution would be needed to look further into this possibility.

### 4.5 Task Performance and fixation frequencies

Fixation frequencies in correct and incorrect trials differed between groups: Neglect patients had the same amount of fixations in both cases; control participants displayed a steep increase in fixations on trials where they apparently lost track of targets; patients without neglect showed a weak increase compared the control group.

If we take rates found in controls as a standard, normal fixation pattern during the MOT task, it would seem that with inefficient tracking, a change in oculomotor strategy is likely to happen. We propose that an observer might go into an overt “search mode,” which may be reflected in an increased fixation rate. Thus, the patterns found in non‐neglect and neglect patients may be triggered from performing poorly on the tasks. A possible floor effect in accuracy scores of neglect patients may have also prevented us to see differences in fixation rates between their correct and incorrect trials.

### 4.6 Limitations

Given that stroke locations of different patients rarely are localized and/or extend over the same areas of the brain, patients often differ with regard to what combination of cognitive deficits they will express. In addition, an individual's health condition or history may influence each patient's outcome and course of recovery differently. Accordingly, generalizing the present findings to the whole neglect or RH population must be done with caution. Considering also that our neglect patients performed poorly in the MOT task, with accuracy scores approaching chance level with multiple targets, the reported differences between correct and incorrect trials must be interpreted cautiously. Finally, our measure of eye movement rates included only eye movements larger than 1° in amplitude, thus excluding microsaccades, glissades etc., which allows generalizations to apply to eye movements with amplitude over 1° only.

The fact that different patients may reveal their neglect symptoms through different types of neglect tests complicates the assessment of neglect symptoms. The BIT‐battery provides a good tool for assessing neglect symptoms as it includes a subset of different tests, which increases the probability that neglect symptoms may be revealed if present. However, there is no guarantee that neglect is reliably revealed by this test battery for all cases, and a possibility remains that some neglect patients, who for example may have learnt to compensate optimally for their deficits in less challenging settings (like the BIT‐assessment) would be wrongly diagnosed as not having neglect. In fact, these patients may show neglect symptoms in more challenging tasks (like the present MOT task). Hence, there is a chance of falsely concluding for the absence of significant differences between these patient groups.

## 5 CONCLUSION

A dynamic divided‐attention task (MOT) combined with recordings of eye movements allow for a simultaneous investigation of several relevant variables for understanding the neglect syndrome. In the present sample of RH patients, performance was dramatically reduced in patients diagnosed with severe spatial attention dysfunction (neglect patients), showing in essence that neglect may be associated with a remarkable deficit in dividing attention into even a few attentional foci. The findings also suggest that an increase in fixations’ rate may be a hallmark of brain (perhaps RH) injury and not only of the presence of severe neglect. However, the neglect patients spatially distributed their gaze rather differently from the other RH patients, and the severity of neglect was predictive of how the distribution of visual fixations was spatially biased in this group. With a few neglect patients showing a spatial gradient in fixations of the opposite direction than that of other neglect patients, this is suggesting the presence of compensatory oculomotor mechanisms in some patients. The dysfunction in orienting of attention in neglect is well described as directional hypokinesia. Increased processing demands when dividing attention between two or three targets may require an increase in eye fixation frequencies.

The findings of this study offer some novel understanding of what mechanisms may be involved in neglect. Specifically, it appears that neglect can be revealed through measures of oculomotor processes such as direction‐specific patterns of eye movement frequencies, even when a patient seems able to complete most of the task successfully. Hence, monitoring oculomotor behavior with an eye‐tracker may provide a very useful tool for assessing attention deficits in different stages of the neglect syndrome and during rehabilitative interventions. Moreover, we have shown in this study that the distribution of fixations across the field may reveal neglect in some but importantly not in all cases of neglect, as possible compensational fixation strategies may mask such symptoms. An increased understanding of the different components that play a role in attention and how resources are allocated in neglect may open for targeted interventions which may focus on measurable oculomotor factors. Additionally, this information could perhaps bring value to clinical assessments of neglect patients in the future and be relevant in estimating the prognosis for recovery or indications of progress during training and treatment. Finally, we can hope that future paradigms based on MOT‐like computerized testing and monitoring of eye movements may contribute with valid and sensitive measurements of neglect severity, targeted rehabilitation to the oculomotor control, as well as a novel way to monitor progress or deterioration in the individual patient over time.
